# Myectomy with and without Mitral Subvalvular Repair in Patients with Hypertrophic Obstructive Cardiomyopathy with Grade 3 to 4+ Mitral Regurgitation without Intrinsic Mitral Valve Disease: A Retrospective Observational Study

**DOI:** 10.31083/j.rcm2308279

**Published:** 2022-08-10

**Authors:** Fangyu Liu, Yulin Wang, Ye Yang, Hao Lai, Kai Song, Chunsheng Wang, Qiang Ji

**Affiliations:** ^1^Department of Cardiovascular Surgery, Zhongshan Hospital, Fudan University, 200032 Shanghai, China; ^2^Department of Cardiovascular Surgery, Xiamen Branch of Zhongshan Hospital, Fudan University, 361015 Xiamen, Fujian, China; ^3^Shanghai Municipal Institute for Cardiovascular Diseases, 200032 Shanghai, China

**Keywords:** hypertrophic obstructive cardiomyopathy, mitral regurgitation, septal myectomy, mitral subvalvular management

## Abstract

**Background:**

Hypertrophic obstructive cardiomyopathy (HOCM) with severe mitral regurgitation (MR) remains controversial for the choice of the concomitant mitral valve (MV) management versus septal myectomy alone. The impacts of different surgical strategies (concomitant mitral subvalvular procedures versus myectomy alone) on one-year results of surgical treatment of HOCM with grade 3 to 4+ MR without intrinsic MV disease were evaluated in this single-center, retrospective observational study.

**Methods:**

A total of 146 eligible patients were retrospectively screened into a combined group (n = 40) and an alone group (n = 106), depending on whether they underwent transaortic mitral subvalvular procedures. Perioperative outcomes were collected, and results at 1-year following surgery were compared.

**Results:**

Surgical mortality did not differ (0 for combined group vs. 0.9% for alone group, *p* = 0.538). Six patients (5.0% vs. 3.8%, *p* = 0.666) developed postoperative complete atrioventricular node block with permanent pacemaker implantation. No death or reoperation was recorded during a median follow-up of 18 months. At 1-year following surgery, (1) the provoked MR severity decreased from baseline in both groups with a significant difference between groups [1.0 (0–1.0) vs. 1.0 (1.0–1.3), *p *< 0.001]; (2) systolic anterior motion (SAM) was observed in 10 patients (0 vs. 10 in the alone group, *p* = 0.043); (3) the provoked gradient was also significantly lower than baseline value for each group, with a significant difference between the two groups (8.8 ± 4.3 mmHg vs. 12.1 ± 6.7 mmHg, *p* = 0.006); and (4) New York Heart Association class decreased from baseline value for each group (*p *< 0.001).

**Conclusions:**

In HOCM patients with grade 3 to 4+ MR without intrinsic MV disease, mitral subvalvular management during septal myectomy may be associated with a low incidence of SAM, improved MR, and a lower outflow tract gradient in comparison with septal myectomy alone.

## 1. Introduction

Hypertrophic obstructive cardiomyopathy (HOCM) is an inherited cardiomyopathy with a prevalence rate of 0.2% globally [1]. It is the most frequent cause of sudden death in athletes [2]. Septal myectomy has become a well-established surgical method for the treatment of symptomatic HOCM patients who require septal reduction therapy [3, 4, 5, 6]. In HOCM patients with concomitant severe mitral regurgitation (MR), some medical centers prefer to perform a myectomy alone, reserving concomitant mitral valve (MV) procedure primarily for intrinsic MV disease [7, 8, 9, 10]. The function of the MV apparatus in the pathophysiology of HOCM has received more attention in recent studies [11, 12, 13, 14, 15]. Several promising additional MV repair techniques, including anterior mitral leaflet extension [16], papillary muscle realignment [17], and edge-to-edge repair [18], have been applied in HOCM patients with severe MR. However, these techniques lack robust clinical evidence and may not be easily reproducible. Surgical management of HOCM with severe MR without intrinsic MV disease remains controversial as to concomitant MV management versus myectomy alone [19].

Systolic anterior motion (SAM) of the MV, which usually appears with MR, is found in the majority of HOCM patients [2]. Since the pathophysiological features of SAM and MR result from the interplay between hypertrophied ventricular morphology, flow vortices within the left ventricle (LV), and the anatomical features of the MV apparatus, a multifaceted strategy for surgery needs to be considered [13]. Mitral subvalvular management during septal myectomy has become an evolving technique in the treatment of HOCM patients who have no intrinsic MV disease [20, 21]. We hypothesize that for therapy of HOCM with severe MR without intrinsic MV disease, concomitant mitral subvalvular management beyond septal myectomy may be associated with favorable results and not be inferior to septal myectomy alone. This study aims to assess the impact of different surgical strategies (transaortic concomitant mitral subvalvular procedures versus transaortic myectomy alone) on short-term results of surgical treatment of HOCM with grade 3 to 4+ MR without intrinsic MV disease. Previous reports have addressed the importance of the treatment of this condition but rarely discussed the surgical procedures performed through the single aortotomy approach, making this single-center experience valuable to provide surgical alternatives for HOCM patients with significant MR.

## 2. Materials and Methods

### 2.1 Patient Characteristics and Treatment groups

From January 2016 to December 2019, all documented HOCM patients over 18 years old of age who met the surgical indications for the treatment of HOCM according to the European Society of Cardiology guidelines [9] at this center were investigated. Inclusion criteria were as follows: (1) grade 3 to 4+ MR accompanied by SAM determined by transthoracic echocardiography (TTE); and (2) undergoing myectomy alone or myectomy plus mitral subvalvular procedures. The MR severity was assessed according to the guidelines of the European Association of Echocardiography [22].

The exclusion criteria included: (1) concomitant intrinsic MV disease (defined as rheumatic, degenerative, infective, mitral annulus calcification, and other organic lesions) requiring MV repair or replacement; (2) having a history of alcohol septal ablation therapy; (3) undergoing simultaneous other valvular surgery; (4) requiring simultaneous coronary artery bypass grafting; and (5) receiving simultaneous Maze IV surgery. This study excluded patients with secondary cardiac hypertrophy induced by hypertension or aortic valvular stenosis. HOCM patients with severe MR without intrinsic MV disease who underwent mitral valve repair or replacement were not included in this study. HOCM patients who underwent transapical myectomy were also excluded from this study.

In this center, surgeons chose to perform a septal myectomy alone or a mitral subvalvular procedure in addition to septal myectomy based on their individual preference. All surgeons were experienced in performing HOCM surgery. The recruited patients were divided into two groups: those receiving mitral subvalvular procedures along with a septal myectomy (combined group) and patients undergoing septal myectomy alone (alone group).

### 2.2 Surgical Procedures

Intraoperative transesophageal echocardiography (TEE) was performed to assess MV structure and function, and to determine structural variations of the mitral subvalvular apparatus and the amount of myocardium to be resected to reduce LV outflow tract obstruction. The aortic valve leaflets were retracted to provide enough space to reach the outflow tract through an oblique aortotomy 7–10 mm above the right coronary ostium. Septal resection was performed using a scalpel starting from the nadir of the right cusp, 5 mm below the aortic valve, and continued leftward to the left trigone. The upper limit of the resection depth was half of the basal thickness. The range of the septal myectomy could be extended beyond the insertion of the papillary muscle and toward the apex of the LV. Mini-invasive operative instruments such as modified long-handled forceps and scissors were routinely utilized for the septal myectomy [23]; which allowed more extensive resection of the myocardium toward the LV apex.

**Fig. 1.**
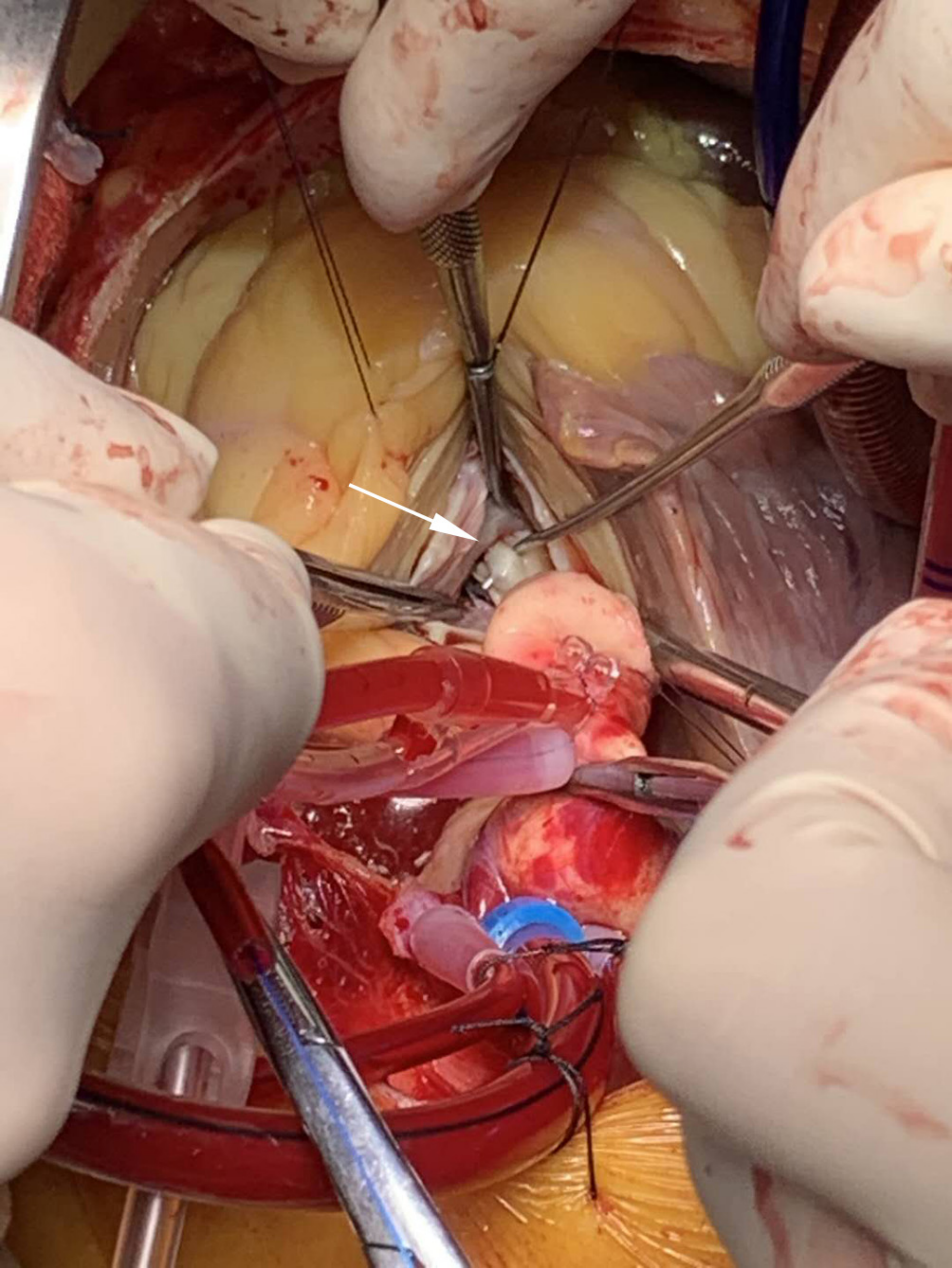
**Intraoperative view of the secondary chordae tendineae**. Transaortic view of preparation for cutting of fibrotic and retracted secondary chordae tendineae inserted on the anterior mitral leaflet body (picked up with a nerve hook, white arrow).

Mitral subvalvular procedures included cutting false cords, papillary muscle release and/or resection, and cutting of secondary chordae. The fibrous or muscular attachments between the septum/free wall and components of the subvalvular apparatus, which became more evident after the septal resection, were separated and excised with sharp dissection. The papillary muscles were inspected to detect any hypertrophy, fusion, displacement, anomalies, or aberrances (e.g., bifurcation or fibrosis). Hypertrophied and thick papillary muscles were split to increase mobility. Accessory papillary muscles were excised. The chordae tendinae were examined to detect any fibrotic, or thickened, secondary chordae tendinae. The abnormal secondary chordae tendinae were resected from the top of the papillary muscle to its insertion onto the anterior MV leaflet (Fig. 1). To avoid iatrogenic MV malfunction, all attachments to the leading edge of the anterior leaflet were preserved.

TEE was used after weaning from cardiopulmonary bypass to evaluate the degree of MR, SAM, and any residual LV gradient, following isoproterenol infusion. Cardiopulmonary bypass was immediately initiated if any of the following abnormalities was observed: residual left ventricular outflow tract (LVOT) obstruction (the provoked gradient of over 30 mmHg), grade 3 or more MR, LV free wall rupture, ventricular septal perforation, or aortic valve perforation.

### 2.3 Study Outcomes

Intraoperative adverse events (including inadequate septal myectomy requiring immediate reoperation, iatrogenic LV rupture, iatrogenic septal perforation, and iatrogenic valve perforation), permanent pacemaker implantation for complete atrioventricular block, surgical death, and any major morbidity were assessed. The STS definitions were strictly followed while the surgical death was determined as death within 30 days of surgery or the same hospitalization, and major morbidity was defined as any occurrence of new-onset atrial fibrillation, new-onset stroke, prolonged ventilation (more than 48 hours or reintubation), renal failure requiring dialysis, deep sternal wound infection, and reoperation for bleeding.

Follow-up data were recorded for further analysis including all-cause survival, MR severity, SAM, LVOT gradient, and New York Heart Association (NYHA) class. Maneuvers such as the stand-to-squat or the Valsalva maneuver were used during follow-up TTE examination. Residual MR was defined as grade 3+ or more MR with provocation determined by TTE examination. Residual LVOT obstruction was determined as the provoked gradient of over 30 mmHg measured by TTE.

### 2.4 Ethics Approval and Consent to Participate 

This is a single-center, retrospective observational study. This observation began in April 2021 and ended in September 2021. This study reviewed the data of eligible patients who underwent septal myectomy with or without mitral subvalvular management in this center between January 2016 and December 2019. Baseline characteristics and perioperative data were retrieved from our electronic hospital database. Patients were routinely followed up at three and six months after surgery, as well as at 6-month intervals after that. The follow-up information was obtained through clinic visits. Telephone interviews and/or short message service were conducted after the commencement of the study. An independent database-monitoring center was invited to double-check the datasets for plausibility. Only completed and verified datasets were used for statistical analysis. All subjects gave their informed consent for inclusion before they participated in the study. The study was conducted following the Declaration of Helsinki, and the protocol was approved by the Ethics Committee of Zhongshan Hospital Fudan University (approval number: B2021-195R).

### 2.5 Statistical Analysis

Statistical analysis was processed with SPSS software version 22.0 (SPSS Inc., Chicago, IL, USA). Categorical data were expressed as frequencies and percentages. Ordinal categorical variables such as NYHA class were compared with the rank-sum test while the other categorical variables were compared between groups using *Fisher’s* exact test or the *Chi-square* test depending on the data property. Continuous variables were shown as the mean ± standard deviation if normally distributed and compared using an independent-samples *t*-test. If non-normally distributed, continuous variables were shown as the median and interquartile range (IQR) and compared with the *Wilcoxon* rank-sum test. A difference would be considered statistically significant at a two-sided *p*-value < 0.05.

## 3. Results

### 3.1 Study Population and Surgical Procedures

**Fig. 2.**
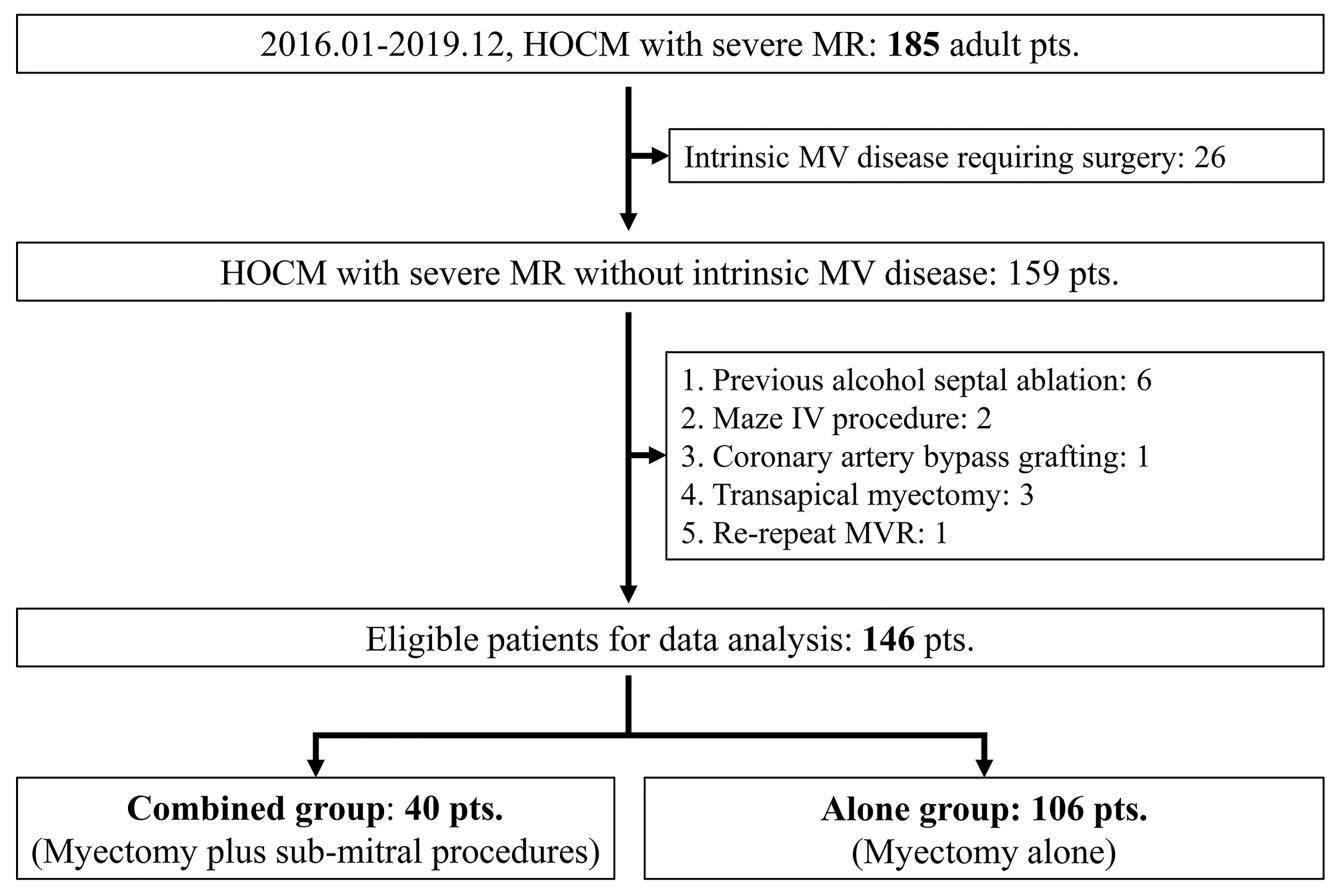
**Flow chart for the selection of the study population**. HOCM, hypertrophic obstructive cardiomyopathy; MR, mitral regurgitation; pts, patients; MV, mitral valve; MVR, mitral valve replacement.

During the study period, 185 adult HOCM patients with grade 3 to 4+ MR without intrinsic MV disease underwent septal myectomy alone or with concomitant mitral subvalvular procedures in this center. As shown in Fig. 2, a total of 146 eligible patients (69 males, with an age of 55.7 ± 11.2 years) with severe symptoms refractory to optimal medical therapy with non-vasodilating β-blockers and/or calcium channel blockers were screened for this study. One patient who underwent immediate re-repeat surgery (MV replacement) due to inadequate initial septal myectomy (septal myectomy plus false cords cutting) was excluded from this study. At baseline, 82.9% of the patients were in NYHA class III or IV. Forty patients underwent transaortic septal myectomy with concomitant mitral subvalvular procedures. The incidence of requiring some type of mitral subvalvular management during the septal myectomy was 27.4%.

Patients were divided into the combined group (n = 40) and the alone group (n = 106), depending on whether they underwent concomitant mitral subvalvular procedures. Patients in the combined group had less septal hypertrophy (septal thickness, 17.1 ± 3.1 mm vs. 18.4 ± 3.3 mm, *p* = 0.033). Demographics, concomitant diseases, preoperative cardiac status, and other echocardiographic data were comparable between the two groups (Table 1).

**Table 1.** **Baseline and surgical characteristics**.

Variables			Combined group	Alone group	*p*
			(n = 40)	(n = 106)	
Demographics					
	Age (years)		53.7 ± 11.4	56.5 ± 11.1	0.179
	Gender (Males)		15 (37.5%)	54 (50.9%)	0.147
	Recent smoking		6 (15.0%)	27 (25.5%)	0.177
Concomitant diseases					
	Diabetes mellitus		2 (5.0%)	9 (8.5%)	0.728
	Hypertension		15 (37.5%)	44 (41.5%)	0.194
	Cerebrovascular disease		1 (2.5%)	2 (1.9%)	>0.999
	Family history of HCM		3 (7.5%)	5 (4.7%)	0.684
Preoperative cardiac status					
	NYHA functional class				0.160
		II	3 (5.0%)	22 (20.8%)	
		III	32 (80.0%)	74 (69.8%)	
		IV	5 (15.0%)	10 (9.4%)	
	Atrial fibrillation		5 (12.5%)	8 (7.5%)	0.344
	Right bundle branch block		1 (2.5%)	3 (2.8%)	>0.999
Echocardiographic data (TTE)					
	Maximum gradients (mmHg)		96.7 ± 23.3	97.0 ± 24.8	0.937
	Septal thickness (mm)		17.1 ± 3.1	18.4 ± 3.3	0.033
	SAM		40 (100%)	106 (100%)	1.000
	MR (median, IQR)		3.5 (3.0–3.5)	3.5 (3.0–3.5)	0.667
	LVEF (%)		66.3 ± 3.9	66.5 ± 4.0	0.871
	LVEDD (mm)		45.2 ± 4.3	44.7 ± 4.4	0.553
Procedures					
	Septal myectomy alone		0	106 (100%)	
	Septal myectomy plus mitral procedures		40 (100%)	0	
		False cords cutting alone	3 (7.5%)		
		Secondary chordae cutting alone	26 (65.0%)		
		Papillary muscle procedure alone	3 (7.5%)		
		False cords cutting + Papillary muscle procedure	4 (10.0%)		
		Secondary chordae cutting + Papillary muscle procedure	4 (10.0%)		
	ACC time (min)		38.0 ± 7.1	35.6 ± 7.3	0.076

HCM, hypertrophic cardiomyopathy; NYHA, New York Heart Association (classification); TTE, transthoracic echocardiography; SAM, systolic anterior motion; MR, mitral regurgitation; IQR, inter-quartile range; LVEF, left ventricular ejection fraction; LVEDD, left ventricular end-diastolic diameter; ACC, aortic cross-clamping.

All patients in the combined group underwent concomitant mitral subvalvular procedures, including secondary chordae cutting in 30 (75.0%) patients, false cords cutting in 7 (17.5%), and longitudinal papillary muscle resection with the mobilization technique in 11 (27.5%). In patients undergoing secondary chordae cutting, 1 to 3 secondary chordae were resected individually based on the anatomy. The combined group had a slightly longer aortic cross-clamp (ACC) time, that was not significantly different (38.0 ± 7.1 mins vs. 35.6 ± 7.3 mins, *p* = 0.076).

### 3.2 Intraoperative Outcomes

As listed in Table 2, intraoperative adverse events were observed in 2 patients from the combined group and 5 from the alone group (*p *> 0.999). In the combined group, one patient developed iatrogenic LV free wall rupture and underwent immediate repeat ACC and LV free wall repair. The other developed iatrogenic aortic valve perforation and received an immediate reoperation (aortic right valve repair). In the alone group, three patients developed insufficient initial septal resection determined by TEE examination and received immediate repeat surgery (“more” extended septal myectomy), and 2 other patients immediately underwent repeat surgery for an iatrogenic LV wall rupture and an iatrogenic septal perforation.

**Table 2.** **Clinical and echocardiographic results**.

Variables			Combined group	Alone group	*p*
Intraoperative			n = 40	n = 106	
	Intraoperative adverse events		2 (5.0%)	5 (4.7%)	>0.999
		Iatrogenic LV free wall rupture	1 (2.5%)	1 (0.9%)	0.474
		Iatrogenic aortic valve perforation	1 (2.5%)	0	0.102
		Inadequate septal myectomy	0	3 (2.8%)	0.282
		Iatrogenic septal perforation	0	1 (0.9%)	0.538
	TEE data				
		Provoked gradients (mmHg)	8.8 ± 5.0	11.5 ± 6.6	0.026
		Septal thickness (mm)	13.5 ± 1.8	14.1 ± 2.1	0.112
		SAM	0	13 (12.3%)	0.020
		Provoked MR severity (median, IQR)	1.0 (1.0–1.0)	1.0 (1.0–1.0)	0.035
In-hospital			n = 40	n = 106	
	Surgical death		0	1 (0.9%)	0.538
	Complete atrioventricular block		2 (5.0%)	4 (3.8%)	0.666
		Previous right bundle branch block	1	2	
	Complete left bundle branch block		20 (50.0%)	37 (34.9%)	0.095
	New-onset atrial fibrillation		2 (5.0%)	3 (2.8%)	0.615
	Cerebrovascular adverse events		1 (2.5%)	2 (1.9%)	>0.999
	Prolonged ventilation (>72 h)		1 (2.5%)	2 (1.9%)	>0.999
	Postoperative hospital stay (d; median, IQR)		6 (5–6)	6 (5–6)	0.218
Follow-up			n = 37	n = 98	
	Follow-up time (m; median, IQR)		15.0 (12.0–18.0)	18.0 (12.0–22.0)	0.116
	Survival with freedom from reoperation		37 (100%)	98 (100%)	>0.999
	TTE data at 1-year following surgery				
		Resting gradients (mmHg)	8.1 ± 4.5	10.8 ± 5.1	0.005
		Provoked gradients (mmHg)	8.8 ± 4.3	12.1 ± 6.7	0.006
		Residual obstruction (Provoked)	0	3 (3.1%)	0.282
		Septal thickness (mm)	13.5 ± 1.8	14.0 ± 2.2	0.219
		SAM	0	10 (10.2%)	0.043
		Resting MR (median, IQR)	1.0 (0–1.0)	1.0 (1.0–1.0)	<0.001
		Provoked MR (median, IQR)	1.0 (0–1.0)	1.0 (1.0–1.3)	<0.001
		Grade 3+ or more MR (Provoked)	0	3 (3.1%)	0.282

LV, left ventricular; TTE, transthoracic echocardiography; SAM, systolic anterior motion; MR, mitral regurgitation; IQR, inter-quartile range; d, day; m, month.

### 3.3 In-Hospital Outcomes

No surgical death was recorded in the combined group; nevertheless, one (0.9%) patient from the alone group died on the fourth day after surgery with a cerebral hernia, which could be related to acute cerebral infarction. Six patients (2 from the combined group vs. 4 from the alone group, *p* = 0.666) underwent permanent pacemaker implantation because of complete atrioventricular node block following myectomy. Five patients (5.0% vs. 2.8%, *p* = 0.615) suffered from new-onset atrial fibrillation but returned to sinus rhythm following electrical cardioversion. As shown in Table 2, other major postoperative complications were comparable between the two groups. In total, 145 patients got discharged with a median postoperative hospital stay of 6 days either for the combined group or the alone group (*p* = 0.218).

### 3.4 Follow-Up Results

A total of 135 patients were followed for a median follow-up of 18 (IQR, 12–22) months. There were no death or reoperations. During follow-up, one asymptomatic ventricular septal defect of 2 mm was observed in one patient from the alone group who was diagnosed with intraoperative iatrogenic septal perforation and underwent repair. The patient is now in NYHA class I.

**Fig. 3.**
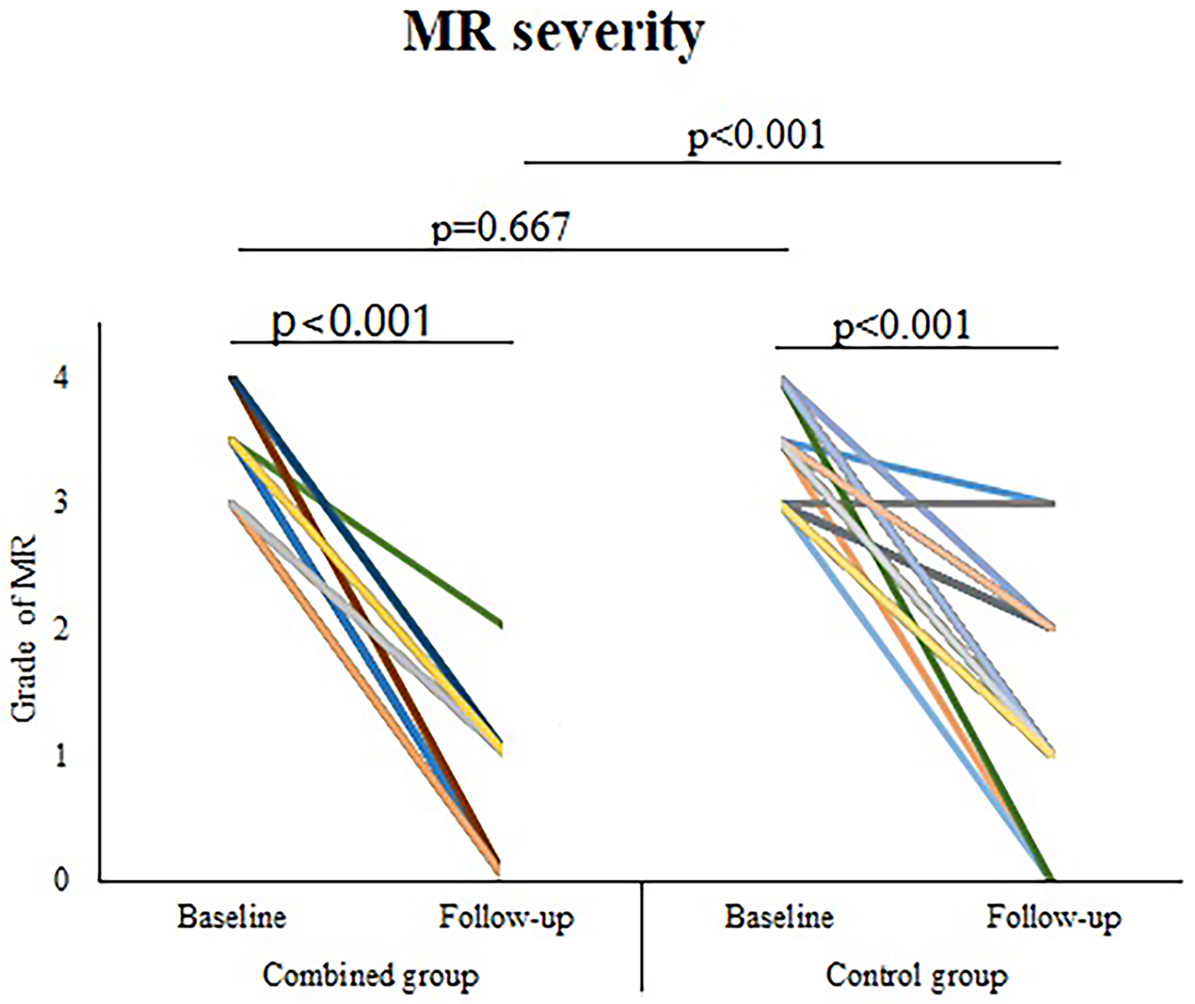
**Provoked MR severity (baseline and 1-year following surgery)**. MR, mitral regurgitation.

**Fig. 4.**
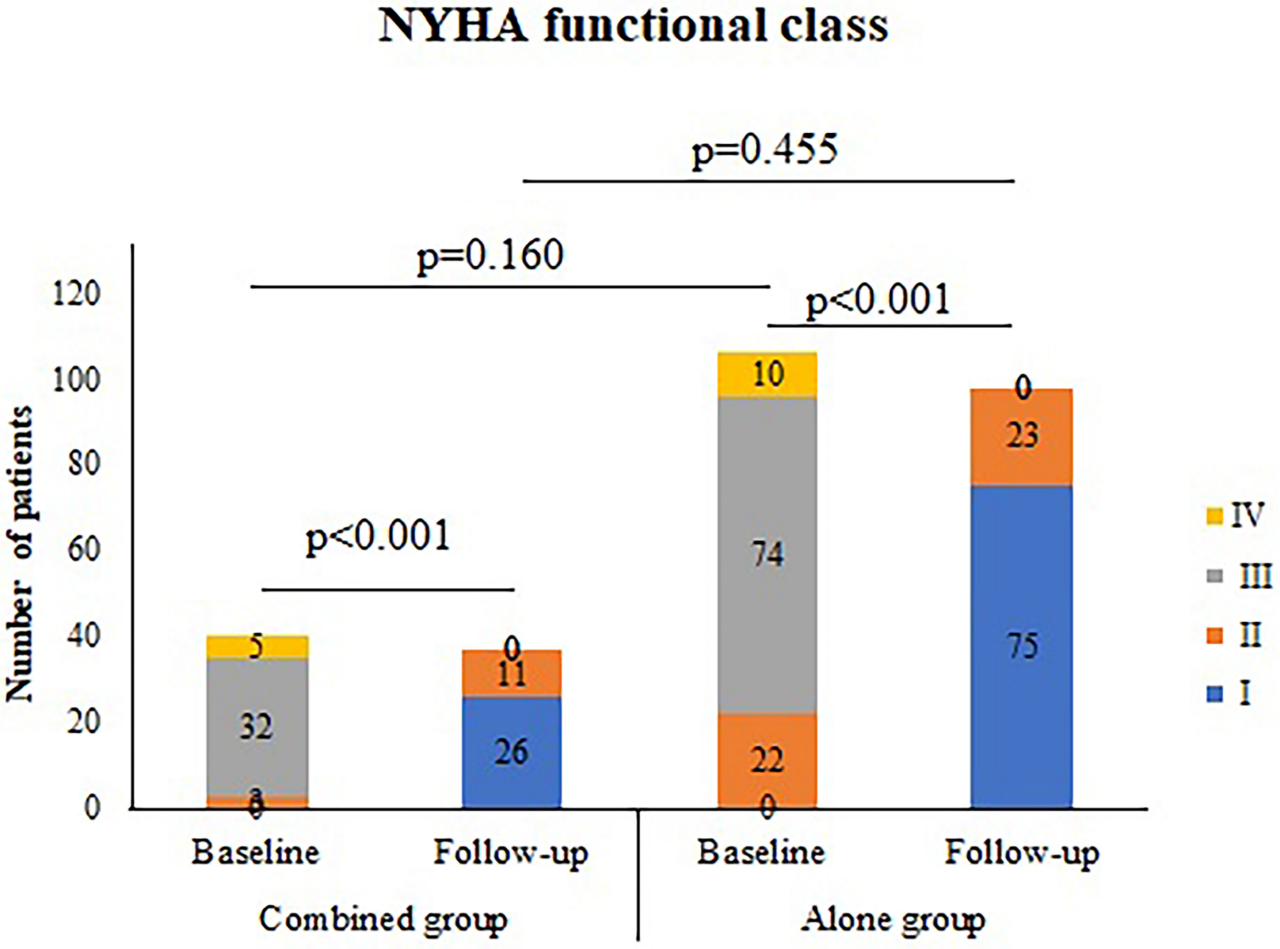
**NYHA functional class (baseline and 1-year following surgery)**. NYHA, New York Heart Association.

At 1-year following surgery, (1) the provoked MR severity decreased from baseline in both groups while with a significant difference between groups (*p *< 0.001) (Fig. 3); (2) 10 patients (all in the alone group) were diagnosed with SAM, including 3 patients with grade 3+ MR and 7 with grade 1 to 2+ MR; (3) no MV flail or prolapse was found following mitral subvalvular procedures; (4) the provoked gradient was significantly lower than baseline in either group, with a significant difference between groups (8.8 ± 4.3 mmHg vs. 12.1 ± 6.7 mmHg, *p* = 0.006); (5) residual obstruction was found in 3 patients from the alone group but not found in the combined group (*p* = 0.282); (6) there was no difference between the two groups regarding septal thickness following surgery (13.5 ± 1.8 mm vs. 14.0 ± 2.2 mm, *p* = 0.219); and (7) as shown in Fig. 4, NYHA class for each group significantly decreased from baseline, which was comparable between groups (*p* = 0.455) with no NYHA III or IV patients.

## 4. Discussion

In HOCM patients, MR may result from SAM or be associated with intrinsic MV disease. In HOCM patients without intrinsic MV disease, the anterior mitral leaflet is pushed into the outflow tract by the flow acceleration along the hypertrophied septum (a “drag” [18] rather than “suck” [24] mechanism), which results in SAM. This is associated with anatomical abnormalities including the elongation of the anterior mitral leaflet, mitral subvalvular abnormalities, anterior displaced hypertrophic papillary muscles, and a too-small distance between the ventricular septum and the anterior mitral leaflet, which may worsen outflow tract obstruction and increase the amount of MR [18, 25]. Mitral subvalvular procedures have been reported to move back the coaptation plane of the MV by freeing the posterior motion of the elongated anterior mitral leaflet away from the septum, thus abolishing SAM and relieving the LVOT obstruction [21]. Therefore, mitral subvalvular management during septal myectomy may provide a potential treatment strategy for HOCM with severe MR in the absence of intrinsic MV disease.

The main finding of this study was that in HOCM patients with grade 3 to 4+ MR without intrinsic MV disease, concomitant mitral subvalvular procedures as compared to myectomy alone were associated with a lower incidence of SAM and an improved MR. Since the SAM in HOCM patients results from the Bernoulli effect, septal thickness following surgery might account for SAM and MR severity regardless of the mitral subvalvular management. In this series, no significant difference between the two groups was found regarding postoperative septal thickness. Therefore, we suspect that the differences in SAM and MR severity postoperatively could not be related to postoperative septal thickness. This study showed that in patients who underwent septal myectomy plus mitral subvalvular management, SAM was abolished, and grade 3 to 4+ MR either decreased to grade 1 to 2+ MR or was completely abolished, regardless of the provoked maneuvers during TTE examination, confirming the benefits of myectomy plus mitral subvalvular procedures on SAM and MR. Patients who underwent combined procedures had a significantly lower incidence of SAM and significantly lower severity of MR with provocation at 1-year following surgery in comparison with patients who underwent myectomy alone, suggesting that concomitant mitral subvalvular management beyond septal myectomy may result in better freedom from SAM and improved MR in comparison with septal myectomy alone. This finding differed from the study by Wei and colleagues, who reported that septal myectomy with concomitant MV procedures resulted in MR reduction similar to septal myectomy alone [25]. The main reason for this difference may be the different study populations, since HOCM patients with intrinsic MV disease were not included in this study.

Another important finding was that septal myectomy with mitral subvalvular procedures was associated with a lower outflow tract gradient compared with septal myectomy alone. This study showed that in each patient who underwent septal myectomy plus mitral subvalvular procedure, the outflow tract obstruction after provocation was completely abolished. This result confirmed the effect of concomitant mitral subvalvular management beyond myectomy on the outflow tract obstruction. Under the condition of comparable baseline gradients, the provoked gradient following concomitant mitral subvalvular procedure was significantly lower than that following myectomy alone. Therefore, this study suggests that the effect of relieving obstruction following myectomy with mitral subvalvular procedures was not inferior to that following myectomy alone, which is similar to a previous study [26].

In this series, a relatively mild degree of septal hypertrophy was found in the combined group in comparison with the alone group, suggesting that in symptomatic HOCM patients with severe MR and only mild septal hypertrophy, mitral subvalvular anomalies are important contributors to MR and concomitant mitral subvalvular management beyond septal myectomy should be considered. Interestingly, there was little difference in the duration of ACC between the two groups. With a relatively thinner septum to deal with, the overall ACC time was only several minutes longer with no statistical significance between the combined group compared to the alone group. This may be more reflective of the more experienced surgical skills of those surgeons who performed the mitral subvalvular procedures in addition to myectomy alone, resulting in some degree of selection bias.

LV free wall rupture, which resulted from the subaortic resection to the MV side, occurred in one patient from the combined group. This life-threatening complication was dealt with by closing the defect using a double-armed 3-0 polypropylene suture with a pledget placed in a horizontal mattress fashion under cardiopulmonary bypass with cardioplegic arrest. Although abnormal papillary muscles were corrected, the LV free wall rupture confirmed at the time of surgery was not thought to be associated with the excision of muscle bundles. The incidence of pacemaker implantation (5.0%) in the combined group was higher than in other reported large series [27, 28]. This could be related to more extensive subaortic septal resection.

The favorable results including significant improvement of MR severity, complete relief of LVOT obstruction, and complete abolishment of SAM coincided with no increase in perioperative adverse events or follow-up mortality, indicating that myectomy plus mitral subvalvular procedures may be an effective treatment strategy for HOCM patients with severe MR without intrinsic MV disease. Therefore, this study supports the application of myectomy plus mitral subvalvular procedures in the treatment of HOCM with severe MR without intrinsic MV disease.

There were some potential limitations in this study. First, this was a single-center, retrospective observational study with a limited sample size, and the results require more statistical verification. Second, a propensity-matching approach may be beneficial for adjusting for differences in baseline characteristics. However, this approach markedly reduces the sample size, especially for this series which included only 40 patients in the combined group and 106 patients in the alone group. The two groups were comparable for baseline characteristics except for septal thickness. The potential bias regarding this important parameter in HOCM might impact the superior results in the combined group. Third, different surgeons in this center could choose to perform myectomy alone or mitral subvalvular procedures in addition to myectomy based on their preference. We can’t rule out the possibility that there were some patients in the alone group with mitral subvalvular abnormality which was not detected by the surgeons because they were not familiar with the combined procedures; which suggests an element of potential selection bias. Finally, mitral subvalvular abnormalities may be underestimated in the etiology of symptomatic HOCM with severe MR, thus suggesting further studies need to be performed to determine its significance.

## 5. Conclusions

In HOCM patients with grade 3 to 4+ MR without intrinsic MV disease, mitral subvalvular management during myectomy may be associated with a low incidence of SAM, improved MR, and a lower LVOT gradient in comparison with myectomy alone.
